# Direct observation of nanowire growth and decomposition

**DOI:** 10.1038/s41598-017-12381-9

**Published:** 2017-09-26

**Authors:** Simas Rackauskas, Sergey D. Shandakov, Hua Jiang, Jakob B. Wagner, Albert G. Nasibulin

**Affiliations:** 1 Department of Applied Physics, Aalto University School of Science, Puumiehenkuja 2, 00076 Aalto, Finland; 2 University of Turin, Department of Chemistry, NIS Interdepartmental Centre and INSTM Reference Centre, Via P. Giuria 7, 10125 Turin, Italy; 3 Kemerovo State University, Krasnaya str. 6, Kemerovo, 650043 Russia; 4 Center for Electron Nanoscopy, Technical University of Denmark, DK-2800 Kgs. Lyngby, Denmark; 5 Skolkovo Institute of Science and Technology, Nobel str. 3, Moscow, 143026 Russia; 6 National University of Science and Technology “MISIS”, Leninsky pr. 4, Moscow, Russia

## Abstract

Fundamental concepts of the crystal formation suggest that the growth and decomposition are determined by simultaneous embedding and removal of the atoms. Apparently, by changing the crystal formation conditions one can switch the regimes from the growth to decomposition. To the best of our knowledge, so far this has been only postulated, but never observed at the atomic level. By means of *in situ* environmental transmission electron microscopy we monitored and examined the atomic layer transformation at the conditions of the crystal growth and its decomposition using CuO nanowires selected as a model object. The atomic layer growth/decomposition was studied by varying an O_2_ partial pressure. Three distinct regimes of the atomic layer evolution were experimentally observed: growth, transition and decomposition. The transition regime, at which atomic layer growth/decomposition switch takes place, is characterised by random nucleation of the atomic layers on the growing {111} surface. The decomposition starts on the side of the nanowire by removing the atomic layers without altering the overall crystal structure, which besides the fundamental importance offers new possibilities for the nanowire manipulation. Understanding of the crystal growth kinetics and nucleation at the atomic level is essential for the precise control of 1D crystal formation.

## Introduction

From the crystal growth fundamentals^1–4^ it is known that typically crystal growth proceeds in four sequences: a) diffusion of the species (atoms, ions, *etc*.) to the growing surface; b) adsorption and desorption of the species onto and from the growing surface; c) adsorbed species diffusion on the growing surface and d) crystal surface growth by incorporating the adsorbed growth species. The adsorption-desorption of the growth species is not only the rate limiting step for most crystal growth conditions, but it also determines the overall crystal evolution to either growth or decomposition.

A nanowire (NW) is an example of an elongated crystal, which preferably grows in one direction. Therefore, it is an ideal object for the investigations of the crystal growth. Among various materials CuO is one of the most attractive model objects, since it is one of the most studied crystals and allows to carry out real time *in situ* observation in a transmission electron microscope. Moreover, the CuO NWs grow by adding atomic layers (ALs) at the tip limited to the growing {111} surface, therefore offering a convenient way for observation of a single AL nucleation and kinetics without a complicated interactions of various crystal orientation^5^.

Thermodynamically, copper oxidation state changes among CuO, Cu_2_O, and Cu as a function of temperature and O_2_ partial pressure^6,7^ with possible pathways of direct reduction (CuO → Cu) or reduction involving either one or two intermediates^8^. Studies of CuO NW reduction were carried out in reducing environments of H_2_/N_2_ plasma^9^, CO^10^ or in vacuum^11^, revealing the CuO-Cu_2_O-Cu transitions. CuO reduction to pure metal without the intermediate oxide was also demonstrated to happen and Cu_2_O phase was shown to form only at special conditions^8,12^. The most of these works relied on XPS or XAES technique observations^13,14^ and allowed to follow the crystal phase or oxidation state changes only *ex situ* and without being able to observe the atomic rearrangement. Therefore, despite the research reported to date, there is still a lack of knowledge about the kinetics of AL transformation and their growth and decomposition mechanisms.

Here, we investigated the crystal growth and decomposition at the atomic scale under respectively oxidative and reductive environments using CuO NW as a model object. To the best of our knowledge, we reported the first clear and atomically resolved transition between the growth and decomposition on the surface of NWs at the AL. We observed a clear AL nucleation character change during the transition from the growth to the decomposition of CuO NWs. Interestingly the layer-by-layer decomposition of NWs happens without disrupting its crystal structure. This study reveals the kinetics and mechanism of the AL arrangement during the crystal growth at oxidative conditions, the decomposition in vacuum and the switch between these two processes. The understanding of the crystal transformations at the AL during the NW growth is crucial to control the NW structure, composition and thereby its properties. The investigations of the CuO NW growth and decomposition are important from the fundamental point of view, as the understanding of the 1D crystal formation mechanism can be extended to other systems.

## Results and Discussion

**Figure 1:**
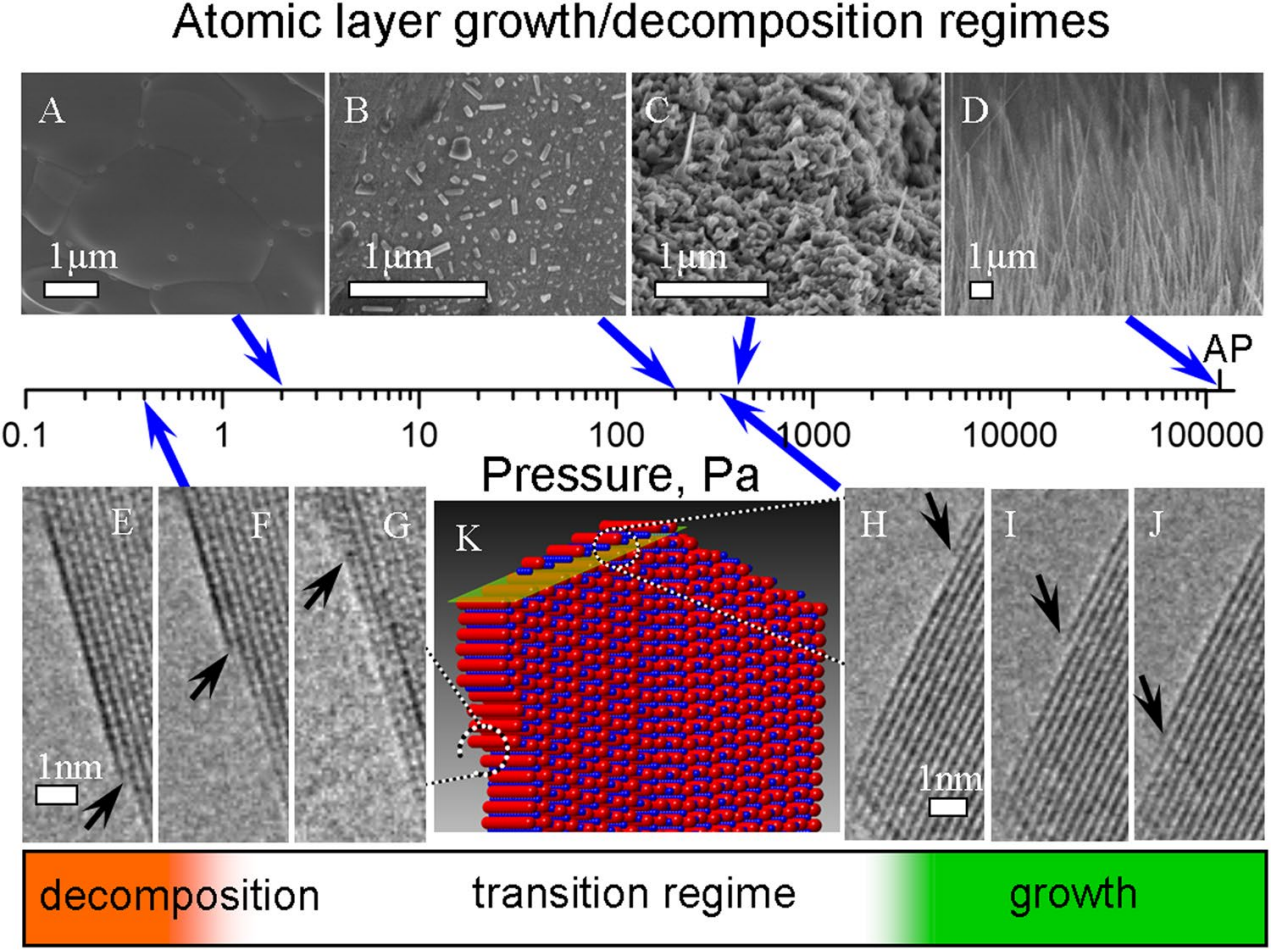
CuO atomic layer (AL) growth/decomposition regimes on NW at different O_2_ pressures and constant temperature of 400 °C. (**A**) to (**D**) are SEM observation of CuO formation at the indicated pressures. *In situ* environmental transmission electron microscopy (ETEM) decomposition of AL (**E**) to (**G**) and AL growth (**H**) to (**J**) were observed on surfaces, schematically shown on CuO NW model (**K**). Atmospheric pressure is indicated as AP on the pressure scale (Supplementary Videos 1–3).

Initially, CuO NW growth was *ex situ* observed by heating Cu samples at the pressure ranging from high vacuum to the atmospheric pressure (Fig. 1). It was found that the NW growth occurs at O_2_ pressures above 400 Pa, and the density of the grown NWs increases along with the pressure. At pressures below 400 Pa, the NW growth was not observed. In the pressure range from 20 Pa to 400 Pa, faceted crystals were formed on the surface (Supplementary Fig. S1). At pressures below 20 Pa no growth of 1D structures was observed, only a planar layer of CuO grew.

**Figure 2:**
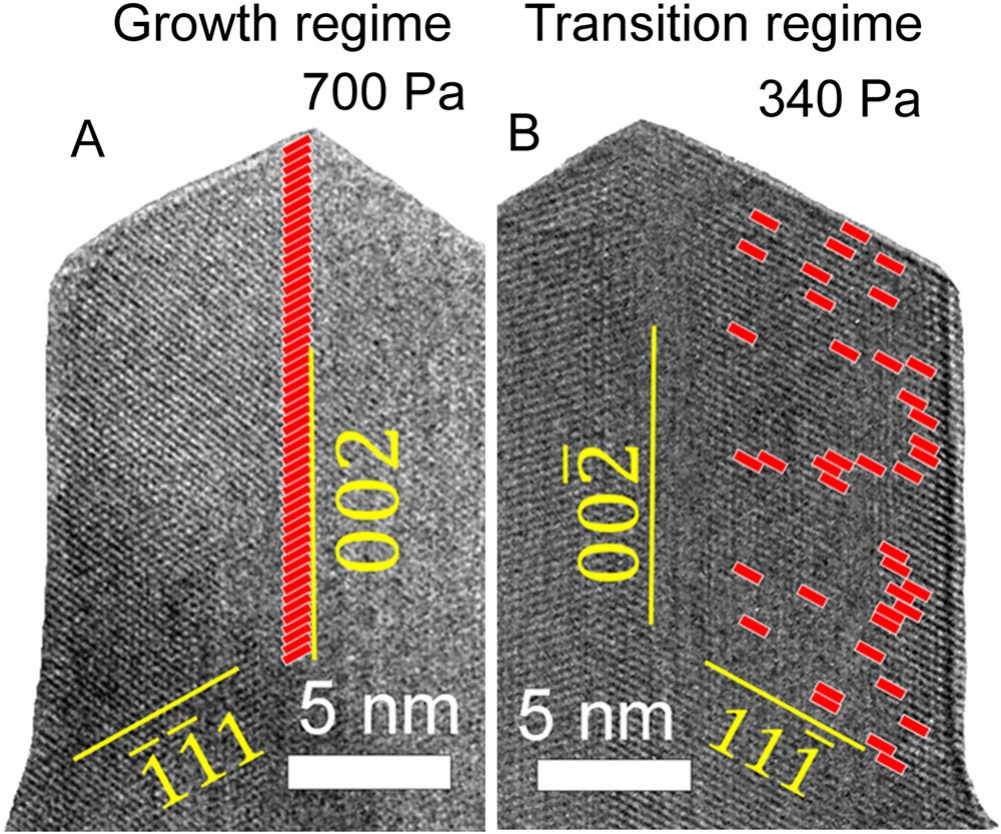
*In situ* observation of CuO NW AL nucleation on {111} surface, nucleation locations of each layer detected during the ETEM observation are marked by red rectangles. (**A**) In the growth regime (at 700 Pa) nucleation can be observed only on the twin boundary, while in the transition regime (**B**) at 340 Pa the nucleation is at the random place on {111} surface (Supplementary Video 1).

The *in situ* environmental transmission electron microscopy (ETEM) observations of CuO NWs under changing O_2_ pressure at a constant temperature (400 °C) revealed 3 AL evolution regimes: the growth, transition and decomposition. First, as it was observed in *ex-situ* experiments, the formation of NW structure, caused by ordered AL growth, was observed only over 400 Pa. The CuO NW growth followed layer by layer manner, when every subsequent AL grows exactly on top of a primary one (Supplementary Fig. S2). ALs nucleated at the edge of the twin boundary ridge, at the tip of NW (Fig. 2A). At the initial O_2_ pressure under 400 Pa, ordered AL growth (NW growth) was not observed, ALs were nucleating randomly and a planar layer of CuO was grown. Interestingly, if the NW formation started at higher pressures (in the growth regime) and the pressure subsequently was reduced during the observation, the ordered growth of ALs proceeded even at lower pressures (from 100 to 400 Pa), even though that ALs nucleate randomly on the growing surface. Figure 2 shows the results obtained during the *in situ* observation of the NW growth initiated at 700 Pa and continued after the O_2_ pressure was reduced to 340 Pa (Supplementary Video S1). The video demonstrates that O_2_ pressure is critical for the initial ordered nucleation of ALs (NW formation), but if the NW structure is already available, ALs nucleate at the growing surface, retaining the ordered structure.

**Figure 3:**
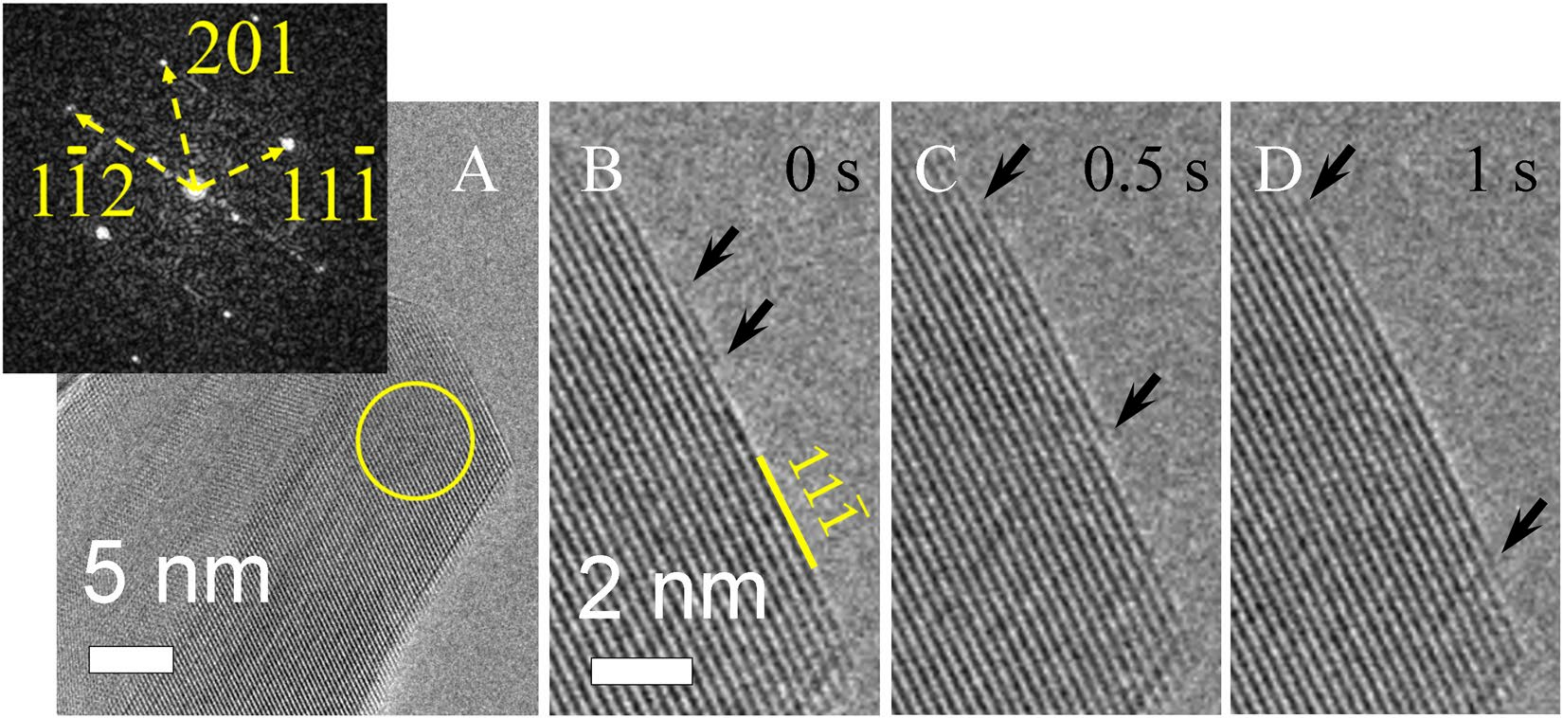
*In situ* observation of CuO NW formation changing from the growth to transition regime, as the initial pressure of 640 Pa was lowered to 340 Pa (Supplementary Video 1). (**A**) is an overview image. FFT shown in the inset was calculated from the circled area; (**B**) to (**D**) show one AL growth progression.

**Figure 4:**
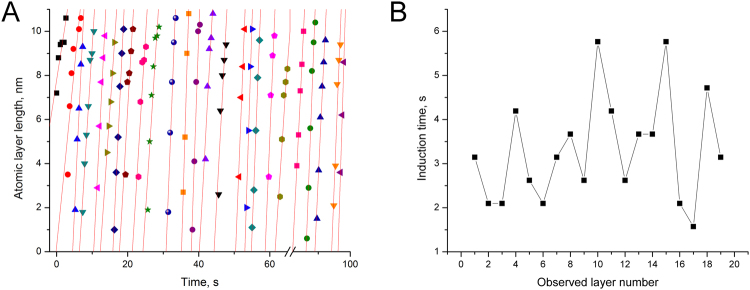
The results of the NW kinetic studies: AL growth parameters in the transition regime. (**A**) time evolution of the NW AL growth rate and (**B**) induction time. Separate ALs in (**A**) are marked with distinctive symbols and lines to guide the eye.

During the formation of ALs in the growth regime, ALs nucleate at the twin boundary of the growing NW (Fig. 2A), however, in the transition regime the AL nucleation is observed at random points on {111} surface (Figs 2B, 3). This shows that the nucleation conditions changed and the twin boundary is not anymore the preferential nucleation point. Interestingly, the AL growth rate 1.7±0.4 nm/s in the transition regime remained in the same order of magnitude as in the growth regime (1.4±0.3 nm/s) even if the O_2_ pressure was lower (Fig. 4). The FFT pattern (Fig. 3A inset) can be well indexed using the monoclinic CuO structure (*a* = 4.69, *b* = 3.43, *с* = 5.13 Å, *β* = 99.55°), which is consistent with our earlier analysis^15–17^.

**Figure 5:**
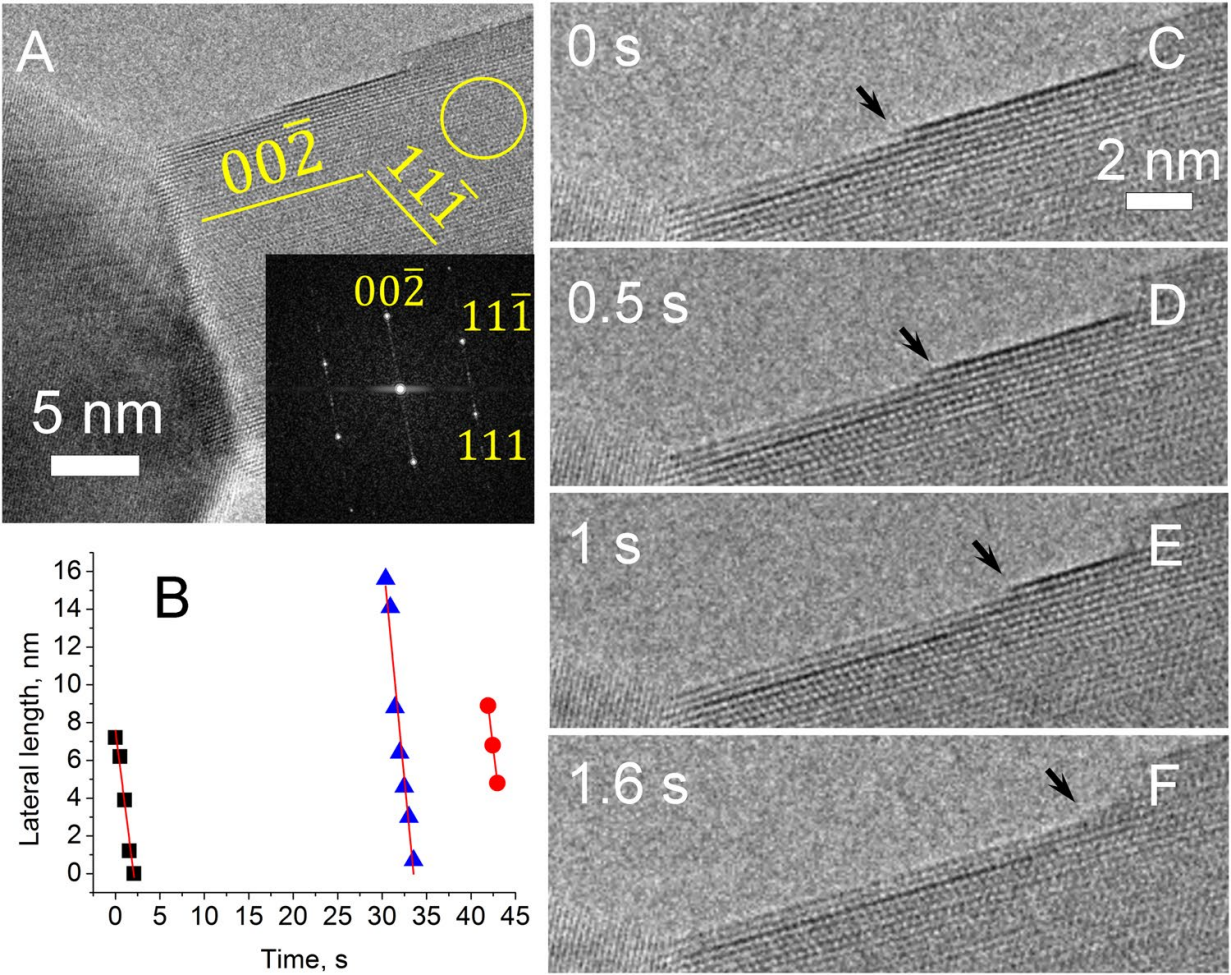
NW transformation in the decomposition regime at 0.4 Pa (Supplementary Video 2); (**A**) shows overview image, inset is FFT from the area marked by a circle; (**B**) graph shows the AL removal rate, separate ALs are marked with distinctive symbols and lines to guide the eye; (**C**) to (**F**) demonstrate the AL removal.

Further decreasing the O_2_ pressure in the system led to the decomposition regime. Experimentally it was found that the decomposition regime occurred at the O_2_ pressures around 0.4 Pa. It can be noticed that the decomposition of NWs takes place in an ordered layer-by-layer manner, similarly to the ordered growth of ALs on the NW tip, but with longer induction time and higher AL decomposition rate of 4.2±0.5 nm/s (Fig. 5). Induction time is a period of time that is necessary to initiate a layer growth, and it was calculated on the basis of ETEM images as the time between nucleation of two consequent ALs.

The decomposition started from the base of the CuO NW on (002) lattice plane. We did not observe the transition in the crystal phase to any intermediates such as Cu_2_O or to metallic Cu. In case of CuO reduction in vacuum, considerable changes can be seen already at 100°C^13^ and the most probable mechanism involves oxygen diffusion to bulk Cu rather than oxygen desorption into vacuum^13,14^. In our case, the decomposition of ALs starts at the base of the NW, which may be related with the shorter diffusion route from the base of NW to the bulk Cu, located underneath the surface (Supplementary Video 2, Supplementary Figure S3).

On the basis of the experimentally determined rate of the AL decomposition (*v*_*dec*_ = 4.2 nm s^−1^, Fig. 5), we can calculate the decomposition time of CuO as *l*/*v*_*dec*_, where *l* = 0.27 nm is the size of CuO molecule, which can be estimated from the bulk density and volume per one molecule (0.021 nm^3^). Thus, we obtain the experimental decomposition time of *τ *= 0.064 s.

We can also estimate the CuO decomposition time based on the decomposition energy, *E*, and the frequency of the CuO lattice vibration, *ν*, as^3^:

1 $$\tau ={\nu }^{-1}exp(E/RT),$$

where *R* is the gas constant, *T* is the absolute temperature. The decomposition energy can be estimated from the formation enthalpy taking into account the excess surface energy on a layer step as *E *= −Δ*H-γl* ^2^/2. Here *γ* is the specific surface energy. Using the numerical values of Δ*H* = −155 kJ mol^−1^, *R* = 8.31 J mol^−1^K^−1^, *T* = 673 K, *ν *~ 10^12^ s^−1^, *γ* = 0.74 J/m^2^ for CuO (111) and *γ* = 0. 86 J/m^2^ for CuO ($\mathop{1}\limits^{\bar{} }$11)^18^ we obtain *τ* *~ *0.06 s and *τ* *~* 0.04 s, respectively. These values are in a good agreement with the decomposition time determined experimentally (0.064 s).

Bearing in mind that during the CuO AL formation two processes – embedding of CuO to the layer and removal of CuO from the layer - are conducted simultaneously. Therefore, let us compare the arrival time of “active” O_2_ molecules (forming CuO), and the removal time of CuO from the layer, defined from eq. (1). The arrival time of “active” O_2_ molecules to the CuO molecule can be estimated similar to Eq. 1 as:

2 $${\tau }_{a}={{\nu }_{g}}^{-1}\exp ({E}_{a}/RT),$$

where *ν*_*g*_ *=* *l* ^2^ *Vn*/4 is a frequency of gas molecule collisions with wall of area *l* ^2^, *V* *=* *(*8*RT*_0_/*πM*_*ox*_)^0.5^ is a mean velocity of O_2_ molecules, *n* = *pN*_*A*_/*RT*_0_ is a number concentration of O_2_ inside ETEM chamber, *p* is an O_2_ pressure, and *M*_*ox*_ is an O_2_ molar weight, *N*_*A*_ is the Avogadro number, *T*_0_ is a temperature of oxygen inside the ETEM chamber, *E*_*a*_ is the activation energy of NW growth. Substituting *E*_*a*_ = 37 kJmol^−1^, *T* = 673 K, and *T*_0_ = 298 K^17^ and the experimental deposition time (*τ*_*a*_ = 0.064 s) to Eq. (2) we obtain *p* = 6 Pa. This means that at the O_2_ pressure lower than 6 Pa the removal from the AL prevails, which explains the experimentally observed decomposition at 0.4 Pa. At the same time, the AL growth is observed at 400 Pa, the pressure which is almost 70 times higher than the pressure above which the CuO formation is preferred. We believe that the excess pressure is related to the formation of the NW, since as we demonstrated above, if the NW structure is already formed, the NW growth proceeds at lower pressure.

It is worth noting that the AL nucleation is usually more favorable at the corner of the facet of the growing crystal, where the supersaturation is higher than at the center (the Berg’s effect)^19,20^. However, as we found from the *in situ* observation, with the pressure decreasing the AL growth may start at any surface point. The nucleation of the AL at random place may be determined by a weakening of the Berg’s effect due to the decrease of CuO molecule concentration gradient along the edge at lower O_2_ pressure.

Thus, the O_2_ pressure affects both interconnected processes: the growth and decomposition. Obviously, there must be such O_2_ pressures at which the 1D growth ceases and at which decomposition prevails. We experimentally found these O_2_ pressures to be around 400 Pa for the AL growth and 0.4 Pa for the decomposition, respectively.

## Conclusions

To summarize, an atomistic examination of the CuO NW evolution in the conditions ranging from oxidative to reductive ones was carried out. The growth, transition and decomposition regimes were experimentally detected and explained from the AL kinetics point of view. The AL growth/decomposition regimes are determined by two simultaneous processes: embedding and removal of CuO from the AL, where both processes are in equilibrium at the O_2_ pressure of 6 Pa. Therefore, the experimentally observed AL decomposition was obtained at pressures below 0.4 Pa as removal process prevails at lower O_2_ pressures. The NW growth is observed at pressures above 400 Pa. The range between those pressures associated with the transition regime, where crystal growth conditions are favorable only in case of previously formed NWs. Moreover, the transition regime is characterized by weakening of the Berg’s effect, which leads to the change of the AL nucleation from the edge of the twin boundary ridge at the NW tip to a random position on the surface at lattice plane {111}.

Decomposition of CuO NW proceeded in ordered layer-by-layer manner at 0.4 Pa from the bottom side of the NW at the lattice plane (002), which can be explained by the shorter diffusion route from the NW base to underlying bulk Cu. A crystal phase transformation from CuO to intermediate Cu_2_O or Cu was not observed.

Although we investigated the AL growth and decomposition only in one kind of NWs, these atomistic principles can be common to the other 1D crystals. Since the decomposition was pronounced in the bottom part of the NW, it can be also used as a method for NW diameter tailoring or removal from the substrate.

## Methods

An aberration-corrected FEI Titan 80–300 ETEM operated at 300 kV was employed for the *in situ* observation of the AL evolution^17,21^. The sample was dispersed in ethanol and drop-casted on a MEMS-based micro-heater from DENSsolutions. After drying at room temperature, the sample was inserted into the electron microscope.

The CuO NWs were synthesized *in situ* in the ETEM by simple oxidation of pure metallic Cu (>99.99%) powder in the presence of O_2_. The decomposition of the NWs was carried out by lowering the O_2_ pressure maintaining the same temperature of the substrate. The NWs were grown directly on the as-received Cu powder, with no patterning or catalyst addition. The observation time at the set temperature was 30–200 min. Typically, the CuO NWs were observed at 400 °C varying the O_2_ partial pressure from 10^–6^ up to 700 Pa. The electron beam dose varied depending on the conditions from 9 × 10^3^ to 9 × 10^4^ electrons/(nm^2^ s). No visible electron beam damage was observed during the *in situ* growth of the NWs, also no visible difference between the observation area and the rest of the sample was found. For the *ex situ* growth of NWs, pure metallic Cu (99.999%) samples, were heated for 30 min in a vacuum tube furnace (inner diameter 50 mm) Carbolite CTF 12/65/550 with built in flowmeter for O_2_ and control unit 3216P1 at O_2_ partial pressures from 20 Pa to the atmospheric pressure. After the synthesis, the samples were transferred directly to scanning electron microscope (SEM, JEOL JSM 7500F) as grown, without any pre-treatment.

## Electronic supplementary material

Supplementary video 1

Supplementary video 2

Supplementary video 3

Supplementary information
