# Supplementary material for: Direct observation of nanowire growth and decomposition
Source: Sci Rep. 2017 Sep 26;7:12310. doi: 10.1038/s41598-017-12381-9 (PMC5615043; doi:10.1038/s41598-017-12381-9)
Supplement: Supplementary file 4 — Supplementary information [file 41598_2017_12381_MOESM4_ESM.pdf]

SUPPLEMENTARY INFORMATION

Direct observation of nanowire growth and decomposition

Simas Rackauskas[1,2], Sergey D. Shandakov[3], Hua Jiang[1], Jakob B. Wagner[4] and Albert G. Nasibulin[5, 1, 6*]*

[1] Department of Applied Physics, Aalto University School of Science, Puumiehenkuja 2, 00076, Espoo, Finland

[2] University of Turin, Department of Chemistry, , Via P. Giuria 7, 10125, Torino, Italy

[3] Kemerovo State University, Krasnaya str. 6, Kemerovo, 650043, Russia

[4] Center for Electron Nanoscopy, Technical University of Denmark, DK-2800 Kgs. Lyngby, Denmark

[5] Skolkovo Institute of Science and Technology, Nobel str. 3, Moscow, 143026, Russia

[6] National University of Science and Technology "MISIS", Leninsky pr. 4, Moscow, Russia

Corresponding Authors: simas.rackauskas@gmail.com, a.nasibulin@skoltech.ru

SUPPLEMENTARY VIDEOS

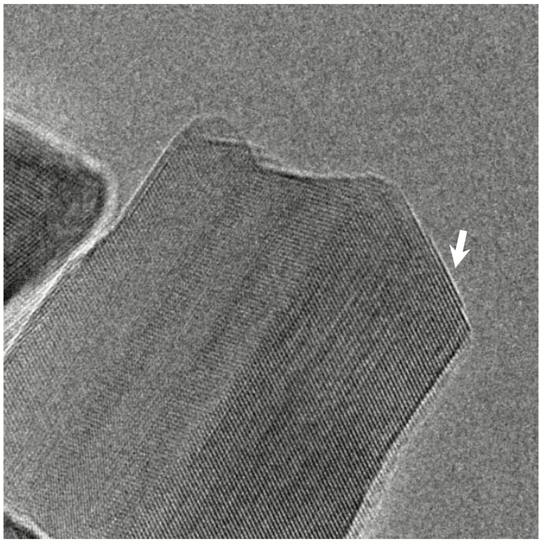

SI video 1. The transition phase: *in situ* environmental TEM movie (MOV) demonstrating the layer-by-layer growth at the tip of NW on lattice plane $(11\bar{1})$. The arrow marks the growth place.

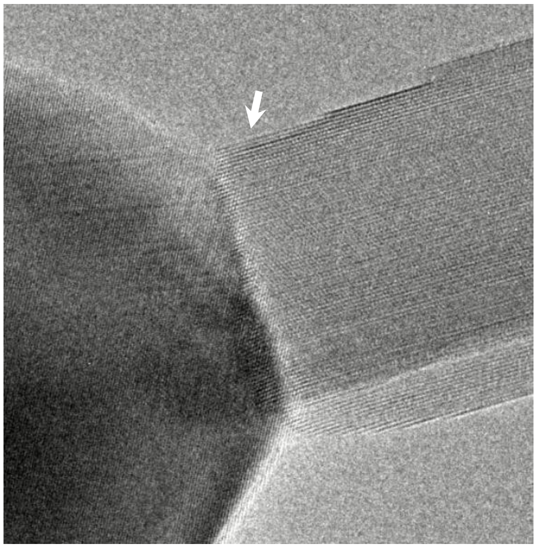

SI video 2. The decomposition phase: *in situ* environmental TEM movie (MOV) demonstrating layer-by-layer decomposition at the bottom of NW on lattice plane $(00\bar{2})$. The arrow marks the decomposition place.

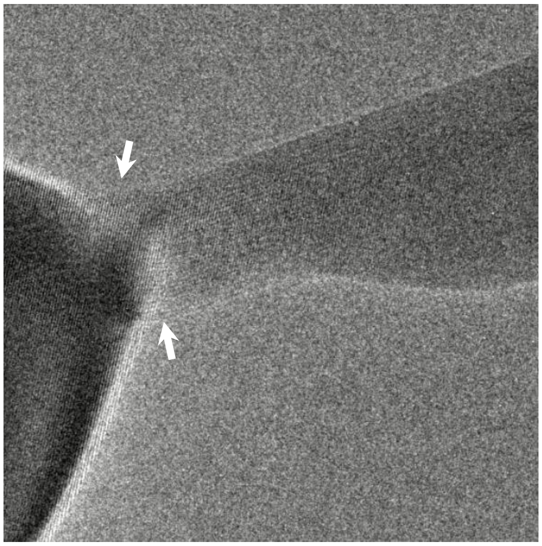

SI video 3. The decomposition phase: *in situ* environmental TEM movie (MOV) demonstrating decomposition and complete NW detachment. The arrows mark the decomposition and detachment place.

FIGURES

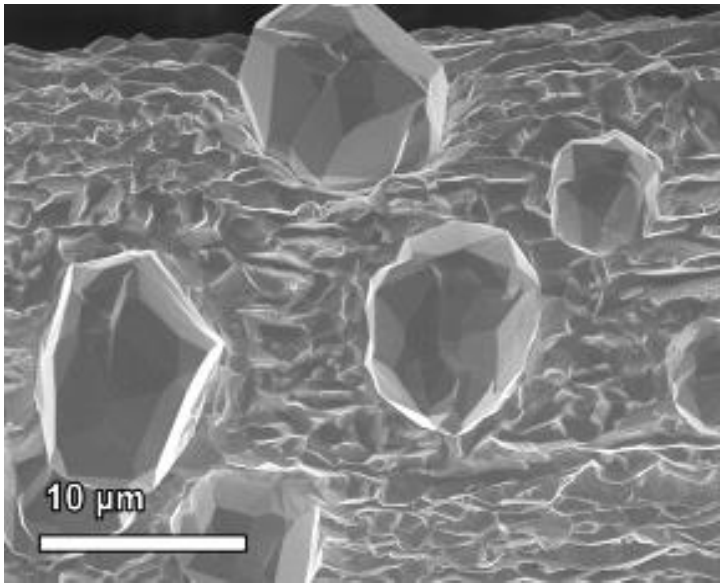

Figure S1. SEM images of structures formed at an O_2 partial pressure of 20 Pa and 400 °C.

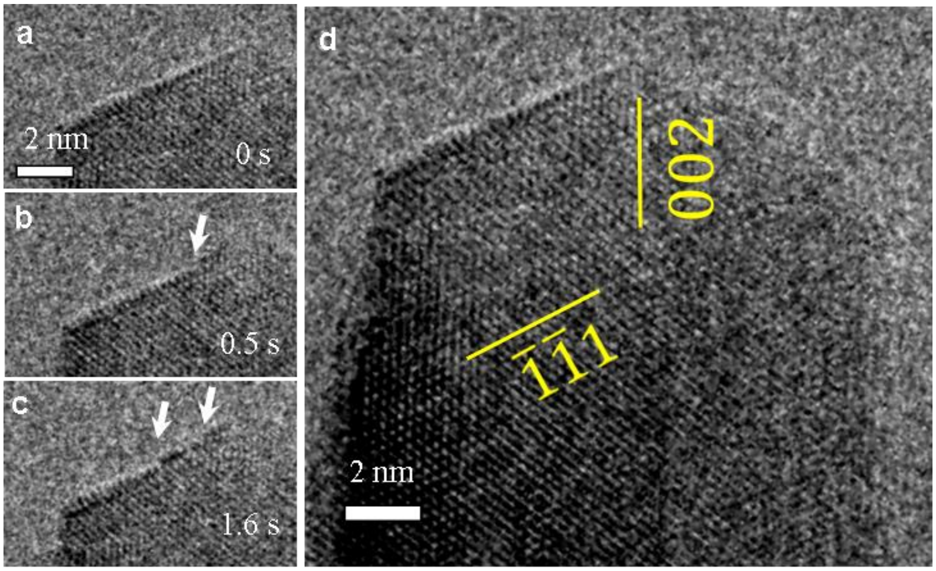

Figure S2. *In situ* environmental TEM images of the atomic layer growth on the tip of CuO NW at 400°C and 700 Pa. (A) to (C) layer by layer growth; (D) overview.

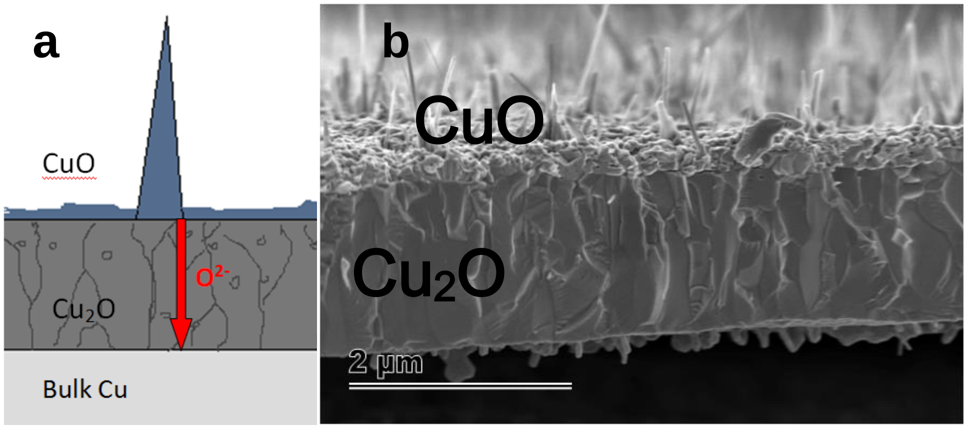

Figure S3. (A) schematics showing oxygen diffusion route from nanowire to underlying Cu bulk. (B) SEM image of the CuO and Cu_2O layers, removed from the surface of the sample after the NW growth.
